# A call to reform medical curricula to sustain the NHS

**DOI:** 10.1080/10872981.2018.1530560

**Published:** 2018-10-16

**Authors:** Priyancaa Jeyabaladevan, Shivaani Yogalingam

**Affiliations:** a Department of Medicine, King’s College London, UK

**Keywords:** Medical, entrepreneurs, curriculum, clinical, innovation

## Abstract

The rise of doctors becoming entrepreneurs can be the solution to the challenges the National Health Service (NHS) currently faces. Doctors and medical students with entrepreneurial aspirations are creating innovative solutions to the problems patients experience. With increasing patient load and decreasing resources in hospitals, it is necessary that medical students are inspired to develop skills that will help to identify problems within organisations and have the knowledge and platforms to convert ideas into reality. By reforming the medical curricula to include health economics and the teaching of successful qualities of entrepreneurship, medical institutes ensure doctors are trained to be drivers of change to sustain the NHS. Instilling an entrepreneurial mindset into a nation of students will nurture forward thinking, innovative students to deliver cutting-edge treatment and care pathways, benefiting both patients and the economy.

Millennial medical students will graduate to become doctors that will experience different challenges and responsibilities to that of the traditional doctor. Each year, 25% of doctors completing foundation training in the UK do not immediately take up specialist training posts and around 5% of junior doctors pursue other opportunities [1]. One of these opportunities is becoming a ‘doctorpreneur’; doctors who are also entrepreneurs. With the reality of an economic crisis encompassing the NHS [2], it is time there was change in the way the doctors of tomorrow are trained. Reforming the medical curricula by incorporating health economics and teaching successful qualities of entrepreneurs, medical institutes ensure doctors are trained to be drivers for change to sustain the NHS and its patients.

Curriculum development is no simple task; however, the advantages of encouraging medical students to turn their ideas into reality will benefit the NHS and the economy.

Nurturing forward thinking, innovative students will bring the benefit of cutting-edge new treatments and care pathways to the patients who need it, whilst stimulating growth within the economy. From our experience as medical students, we are already developing skills that can be transferred to entrepreneurship such as critically appraising evidence, diagnosing problems, communicating effectively, and analysing and interpreting data. Enhancing the curriculum will involve supplementing an education of the commercial world. Medical students should be exposed to commercially relevant knowledge via entrepreneurial online seminars, workshops, and mentoring from successful entrepreneurs. Furthermore, recognised accredited modules, master’s degrees, and intercalated qualifications can be offered to keen students. Interestingly, similar models have been proposed in some universities, but not executed on a national scale [3].

In November 2015, NHS England opened a national training programme: Integrated Clinical Entrepreneur Training, which recruits clinicians with an interest in innovation [4]. Though this programme is remarkable in that it provides a national platform for clinicians to network and gain exposure, the impact of this concept would be more extensive if such mentorship was introduced at an earlier stage. Providing a nation of students with a curriculum that teaches to identify and resolve deficits in systems, will lead to an influx of innovative solutions to the challenges the NHS currently faces.

Despite all the benefits, there are issues that need to be addressed. In general, doctors are embedded with the traditional ethos of medical professionalism, which includes values such as autonomy, beneficence, non-maleficence, and justice [5]. In contrast, the ethos of business-like health care focuses on values such as efficiency, performance, and quality indicators [6]. These differing values could compete for dominance, leading to flawed business models. To limit this, it is imperative to teach the balance between the ethics of medicine and business.

The current climate of the NHS deals with providing health care for an increasing number of patients with a decreasing number of hospital resources. We believe the resulting strain put on health care professionals can be combatted by innovation and entrepreneurship. Medical curricula need reform with the goals of instilling an entrepreneurial mindset in all graduates. Every threat to the existing practice of medicine is an opportunity for doctor entrepreneurs to create solutions.
